# Developing an Internet-Based Cognitive Behavioral Therapy Intervention for Adolescents With Anxiety Disorders: Design, Usability, and Initial Evaluation of the CoolMinds Intervention

**DOI:** 10.2196/66966

**Published:** 2025-04-08

**Authors:** Nikita Marie Sørensen, Helene Skaarnes, Kim Mathiasen, Mikael Thastum, Johanne Jeppesen Lomholt

**Affiliations:** 1 Department of Psychology and Behavioral Sciences Aarhus University Aarhus C Denmark; 2 Department of Clinical Research University of Southern Denmark Odense Denmark; 3 Centre for Digital Psychiatry Mental Health Services Region of Southern Denmark Odense Denmark

**Keywords:** user-centered design, digital treatment, digital mental health, internet-based, cognitive behavioral therapy, anxiety, adolescents

## Abstract

**Background:**

Digital mental health interventions may help increase access to psychological treatment for adolescents with anxiety disorders. However, many clinical evaluations of digital treatments report low adherence and engagement and high dropout rates, which remain challenges when the interventions are implemented in routine care. Involving intended end users in the development process through user-centered design methods may help maximize user engagement and establish the validity of interventions for implementation.

**Objective:**

This study aimed to describe the methods used to develop a new internet-based cognitive behavioral therapy intervention, CoolMinds, within a user-centered design framework.

**Methods:**

The development of intervention content progressed in three iterative design phases: (1) identifying needs and design specifications, (2) designing and testing prototypes, and (3) running feasibility tests with end users. In phase 1, a total of 24 adolescents participated in a user involvement workshop exploring their preferences on graphic identity and communication styles as well as their help-seeking behavior. In phase 2, a total of 4 adolescents attended individual usability tests in which they were presented with a prototype of a psychoeducational session and asked to think aloud about their actions on the platform. In phase 3, a total of 7 families from the feasibility trial participated in a semistructured interview about their satisfaction with and initial impressions of the platform and intervention content while in treatment. Activities in all 3 phases were audio recorded, transcribed, and coded using thematic analysis and qualitative description design. The intervention was continuously revised after each phase based on the feedback.

**Results:**

In phase 1, adolescent feedback guided the look and feel of the intervention content (ie, color scheme, animation style, and communication style). Participants generally liked content that was relatable and age appropriate and felt motivating. Animations that resembled “humans” received more votes as adolescents could better “identify” themselves with them. Communication should preferably be “supportive” and feel “like a friend” talking to them. Statements including praise—such as “You’re well on your way. How are you today?”—received the most votes (12 votes), whereas directive statements such as “Tell us how your day has been?” and “How is practicing your steps going?” received the least votes (2 and 0 votes, respectively). In phase 2, adolescents perceived the platform as intuitive and easy to navigate and the session content as easy to understand but lengthy. In phase 3, families were generally satisfied with the intervention content, emphasizing the helpfulness of graphic material to understand therapeutic content. Their feedback helped identify areas for further improvement, such as editing down the material and including more in-session breaks.

**Conclusions:**

Using user involvement practices in the development of interventions helps ensure continued alignment of the intervention with end-user needs and may help establish the validity of the intervention for implementation in routine care practice.

## Introduction

### Background

Digital mental health interventions (DMHIs) may help increase access to psychological treatment as provision via the internet is presumed to counter some of the key treatment barriers, such as social stigma [1,2], preference for self-reliance [1,2], treatment costs [3], and waiting times [2,3]. It is also well documented that internet-based cognitive behavioral therapy (ICBT) with support from a therapist (eg, via telephone or email) is effective for a variety of mental health problems, including anxiety in children and adolescents [4-7].

However, many evaluations of ICBT programs for children and adolescents with anxiety report high rates of dropout and that participants often fail to complete assessments or treatment sessions [8-12]. In addition, when transitioning to routine care settings, these challenges with adherence and engagement remain [13].

It is still unclear whether participants drop out of treatment due to symptom improvement, symptom deterioration, or other reasons outside therapy. However, working experts in the field of ICBT for children and young people propose that the use of user-centered design methods may help maximize user engagement [14,15]. This methodology emphasizes the importance of (1) understanding adolescents as experts on their own preferences and (2) enabling adolescents to hold central positions as experts in all stages of the design process. As it was eloquently stated in a qualitative study of the Australian Momentum platform, this type of design process may be characterized by “a *designing with* as opposed to a *designing for* mindset” [16].

User-centered design processes typically involve three phases of development: (1) identifying needs and design specifications (with end users), (2) designing prototypes of the intervention and testing their usability, and (3) running feasibility tests with end users [17,18]. The design process must be understood as iterative, and each phase can be revisited if needed as the data collection and analyses progress.

To the best our knowledge, only a few studies describe the inclusion of end users in the design of ICBT interventions for children and adolescents with anxiety [16,19,20]. Hill et al [20] and Ludlow et al [16] both used co-design practices involving service users (parents and children) and service providers (clinicians from routine care) in the development of their ICBT anxiety interventions. In an initial evaluation of the clinical effectiveness, Hill et al [21] found that the dropout rate for their intervention was lower (13%) than those reported in other studies conducted in routine care (32% in the study by Moor et al [22], 21% in the study by Waite et al [13], and 17% in the study by Vigerland et al [23]). This indicates that using user-centered design practices may result in better adherence and engagement with the intervention when delivered in routine care settings.

The importance of including end users in the design of DMHIs has been increasingly acknowledged as imperative not only in developing DMHIs but also in the processes of implementing the interventions [14]. The project that this paper describes was initiated by the Region of Southern Denmark, a government organization that is responsible for the provision of health care services for the residents of the southern municipalities in Denmark. This was done with an intention of broadening the service provision of a DMHI to routine care settings at a national level. Transitioning from research to routine care settings can be tricky, and evidence from clinical trials alone does not guarantee the use of an intervention in routine care. Using user-centered design practices in the development of this intervention may facilitate a smoother transition to large-scale service provision.

### Objectives

The objective of this study was to describe the methods used to develop a new ICBT intervention for adolescents aged 12 to 17 years with clinical anxiety disorders, CoolMinds, within a user-centered design framework.

Our hope is that the methods and results described in this paper will provide inspiration for others on how to include end users in the design processes of DMHIs. In this study, we specifically collected data focusing on how to enhance engagement with the DMHI guided by the following objectives:

1. What are adolescents’ preferences on graphic identity and communication styles within a digital intervention?
2. How do adolescents prefer to seek help and be supported by their parents in this?
3. How do adolescents navigate a prototype of the digital platform and content?
4. What are the families’ initial impressions of the platform and content while in treatment?

## Methods

### Setting

Intervention development was initiated in January 2022 as a collaborative project involving the Centre for the Psychological Treatment of Children and Adolescents and the Centre for Digital Psychiatry. The Centre for the Psychological Treatment of Children and Adolescents is a research center nested within the Department of Psychology and Behavioural Sciences at Aarhus University and is specialized in studying and delivering anxiety interventions to children and adolescents. The Centre for Digital Psychiatry is a research, development, and treatment facility specialized in the use of digital technologies for health promotion and psychiatric treatments and is located within the Mental Health Services in the Region of Southern Denmark.

The project includes intervention development, a feasibility trial, and a randomized controlled trial (RCT). In this paper, only design processes and results from intervention development and the evaluation of the first intervention prototype in the feasibility trial are presented.

### Ethical Considerations

The feasibility trial and RCT were ethically approved by the Medical Research Ethics Committees in Denmark (ID: VMK 2211954), and the protocol for these studies was preregistered in ClinicalTrials.gov (NCT06076964) and published in *Trials* [24]. The workshops and usability tests were conducted as development projects, which do not require ethics approval. The reason for this is that these activities did not include any experimental manipulations, were not targeted at a vulnerable population, and the potential risks associated with participation in these activities were evaluated as very low. Participants were not compensated for taking part in any of the activities or studies.

For the workshops and usability tests, written informed consent was obtained regarding participation, audio recordings, and publication of results. For adolescents aged <15 years, consent was obtained from parents. For adolescents aged ≥15 years, consent was obtained from the adolescents themselves. However, parents were informed of the adolescents’ participation and were urged to discuss this with their children before participation.

For the feasibility trial and the RCT, written and verbal informed consent were obtained from all parents regarding participation and publication. In addition, verbal informed consent was obtained for adolescents aged <15 years, and both verbal and written informed consent were obtained for adolescents aged ≥15 years.

An additional written consent form was used to obtain consent regarding participation in, recording of, and publication of information from feasibility interviews. For adolescents aged <15 years, written informed consent was obtained from parents. For adolescents aged ≥15 years, written informed consent was obtained from both the adolescents and parents.

The quotes reported in this paper were translated from Danish into English. Quotes containing sensitive or identifying information were excluded to protect participants’ anonymity.

### Procedure

The CoolMinds ICBT intervention was developed and continuously updated from January 2022, when the initial outlining of the project began, until June 2023, when the feasibility trial concluded. The development of the intervention followed the recommendations from Hill et al [14] and progressed in three iterations following the design phases: (1) identifying needs and design specifications, (2) designing and testing prototypes, and (3) running feasibility tests. Reporting of the study followed the Guidance for Reporting Involvement of Patients and the Public, and a Guidance for Reporting Involvement of Patients and the Public long-form checklist is available in Multimedia Appendix 1 [25].

### Working Groups

Development processes were led by a working group consisting of junior researchers (authors NMS and HS); a psychologist with experience in producing written materials for adolescents (see the Acknowledgments section); and members of the development and implementation team, such as graphic designers, user involvement experts, and engineers (see the Acknowledgments section) and were supervised by senior researchers (authors JJL, KM, and MT).

During the development process, weekly meetings were held with all available team members. The meetings always commenced with a brief status from all involved teams. The purpose of these meetings was to facilitate the decision-making and implementation processes by assembling relevant team members [26]. This ensured a flow of information between teams when decision-making required the expertise of multiple disciplines (eg, the psychological content had to be informed by data from the user involvement practices and be aligned with the technical possibilities within the platform). When consensus on each topic was reached by all teams, smaller working groups were formed with relevant team members who implemented the decision-based outcomes.

Furthermore, a total of 3 team workshops were held with members representing each team in the working group. The workshops were held at different time points in the development process reflecting the writing of the intervention manuscript. The purpose of the workshops was to (1) map out the overall framework of the intervention content and (2) become aware of any missing information that would need to be researched or collected using user involvement practices. The topics of these workshops were (1) outlining the intervention content, (2) rethinking how to conduct exposure therapy in a digital format, and (3) parental involvement.

### Outlining the Program

The outline of the program was drafted by the working group with expert supervision. This included decisions on therapeutic approach, key treatment components, and parental involvement. It was decided to develop a cognitive behavioral therapy (CBT)–based program as this is the most well-researched approach and has shown good results in previous evaluations using the internet-based format [27-31]. Decisions on which treatment components to include were based on either evidence from research and literature or clinician experience. Several reviews and meta-analyses suggest that key CBT components such as exposure therapy (in vivo and imaginal) and cognitive restructuring are some of the main components driving the treatment effects [27,32,33]. Evidence on the efficacy of coping strategies is scarce compared to that for the key components [34]. However, the group of clinicians involved in providing feedback on the program manuscript advocated for the inclusion of coping strategies as these, in their experience, were techniques that adolescents usually benefitted from and were easier for them to implement on a day-to-day basis. This is also in line with findings from qualitative evaluations of ICBT treatments, where adolescents preferred easy-to-use techniques [35,36]. It was also decided to create a separate parent program and offer the treatment as a family-based intervention as social support from parents may increase treatment adherence and response when treatment is delivered in a digital format [37]. However, the 2 programs were not connected on the platform, and thus, adolescents and parents were allowed to progress at their own pace within each of their programs.

In both programs, sessions were structured in a predefined order starting with psychoeducation followed by cognitive restructuring techniques and, finally, exposure therapy techniques. Sessions were opened automatically upon completion of the previous session unless the adolescent was to receive feedback from a therapist. In that case, the session was first opened upon receiving feedback.

**Figure 1:**
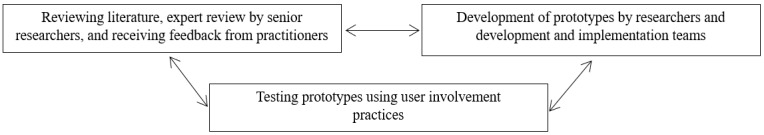
Model of the iterative design process used across phases of development.

Writing of the program manuscript was guided by experiences from previous and linked research activities, current literature in the field, and expertise of senior researchers and experienced clinical psychologists within and external to the project who continuously provided feedback on the manuscript. The literature and practitioner expertise were revisited during the development process to qualify and provide context to the incorporation of insights from the user involvement workshop and usability tests conducted using prototypes of the intervention. This iterative design process is illustrated in Figure 1.

### Phase 1: Identifying Design Specifications in a User Involvement Workshop

A total of 24 adolescents in grade 9 (aged >15 years) from a Danish public school participated in a user involvement workshop held in September 2022. The purpose of the user involvement workshop was to identify adolescents’ preferences on graphic identity and communication styles within the intervention content (objective 1) and investigate adolescent help-seeking behavior (objective 2).

Activities included (1) voting for their favorite prototypes (out of a selection of 3-5 prototypes) using stickers, (2) discussing the pros and cons of each prototype in smaller groups and plenary, and (3) discussing how to reach out for help. Prototypes included different color schemes, styles of animation, expert videos, and types of feedback from the app (push notifications, mood ratings, and in-app rewards and praise). For polls including many prototypes (ie, color schemes, animation style, and notifications), adolescents had more than 1 vote. However, they were also allowed not to use all their votes. The topic of reaching out for help and receiving support was discussed in a column exercise in which adolescents were to give advice to another adolescent in mock-up scenarios about (1) how to handle parents being overinvolved, (2) how to involve parents, and (3) how to handle not having parental support and where to go for help then. Their advice was written down by an assistant in each group. Outcomes of the workshop were documented by audio recording group discussions and through photographs of votes and plenary summaries (both done on a whiteboard).

Members of the development team contacted 4 public schools via phone and, if they were interested, provided them with written information about the content and purpose of the workshop. One school agreed to participate, and the time of the workshop was planned in collaboration with the grade 9 teacher. The written information was then shared with parents via the school’s intranet. Informed written consent was obtained from all adolescents at the beginning of the workshop using a printed consent form.

### Phase 2: Testing Intervention Prototypes in Usability Tests

In total, 4 adolescents (all female) participated in individual usability tests during November 2022 and December 2022. A total of 2 tests were conducted in person with 50% (2/4) of the adolescents, who were aged 12 years, and 2 tests were conducted online with 50% (2/4) of the adolescents, who were aged 16 years. The purpose of the usability tests was to understand how adolescents would navigate the digital platform and perceive the intervention content within this platform (objective 3). Participants were instructed by an assistant to go through a psychoeducational session of the program and say out loud what they thought they were to do in the program and what they then did. They were also prompted to point out anything that confused them and anything that they were particularly fond of in the program. The assistant was present during the entire usability test and took extensive notes used for briefly interviewing the participants about their experience with the program content (eg, animation style and intelligibility of the text) and interface (eg, intuitiveness of navigating the platform), along with some standardized questions (Multimedia Appendix 2). The usability tests were also audio recorded.

Participants were recruited through word of mouth by members of the research group. Informed written consent was obtained from caregivers to children aged <15 years using a printed consent form. Informed written consent was obtained from adolescents aged >15 years using a printable digital consent form sent and received using secure emails (ie, encrypted and digitally signed emails).

### Phase 3: Feasibility Tests With End Users

A total of 7 families (adolescents and their parents) from the feasibility trial participated in a 2-part online interview regarding (1) their satisfaction with trial procedures and (2) their experiences using the program and with specific content components. We developed a semistructured interview guide with open-ended questions regarding general topics related to the trial and treatment procedures and questions regarding specific components of the program content (Multimedia Appendix 2). In this paper, only results regarding the program content are elaborated on. Results on satisfaction with trial procedures will be published elsewhere.

The purpose of these interviews was to explore the families’ initial impressions of the platform and content while still in treatment (objective 4). The interviews were conducted with each family individually but at different time points during their treatment period. The families were picked at random during March 2023 and April 2023 depending on their progress (ie, whether they were at the beginning of, midway through, or at the end of their treatment period). This was done to ensure the collection of information about session-specific content while it was still novel for the participants. The interviews were conducted online and recorded.

For the participating adolescents (n=7), the mean age was 13.29 (SD 1.38; range 12-16) years. A total of 57% (4/7) were male, and 43% (3/7) were female. In total, 43% (6/14) of the parents had a high school or vocational education, 50% (7/14) had completed medium-cycle higher education, and 7% (1/14) had completed long-cycle higher education. All adolescents and their parents were born in Denmark.

Informed written consent was received from all participants aged >15 years and their caregivers before participation using an online consent form distributed using REDCap (Research Electronic Data Capture; Vanderbilt University), a secure, web-based software platform designed to support data capture for research studies [38,39] hosted at the Open Patient Data Exploratory Network within the Region of Southern Denmark.

### Data Analysis

#### Phase 1: Identifying Design Specifications in a User Involvement Workshop

Votes were manually counted and ranked from the highest to lowest number of votes. The audio files from group discussions on pros and cons were transcribed, and the main arguments for and against each prototype were listed by prototype.

Audio files on help-seeking behavior were transcribed and analyzed using a qualitative description design by author NMS [40]. Data were analyzed using content analysis, and codes were sorted into themes.

#### Phase 2: Testing Intervention Prototypes in Usability Tests

Notes from the usability test were analyzed using a qualitative description design [40]. Data were coded into predefined categories reflecting each feature assessed in the usability test (eg, text, video, task, and animation).

Audio files were only used to expand the notes if anything was unclear.

#### Phase 3: Feasibility Tests With End Users

Due to technical issues, recordings from only 71% (5/7) of the interviews were available. These interviews were transcribed and analyzed using a qualitative description design by author NMS [40]. Data were coded using content analysis based on features (eg, text, video, animation, and audio) and preferences (ie, likes or dislikes). Data were then sorted to identify similar patterns, commonalities, and differences. The codes were then grouped into two main themes representing the general feedback from the participants on (1) satisfaction and acceptability and (2) parental involvement, as predefined in the interview guide.

## Results

### Phase 1: Identifying Design Specifications in a User Involvement Workshop

#### Overview

Results from the user involvement workshop are reported in Table 1.

**Table 1:** Overview of adolescent votes and comments from the user involvement workshop conducted at a Danish school, divided into pros and cons. Note that some statements are summaries of participants’ comments and some are direct quotes (indicated by quotation marks).

			Number of votes		Pros		Cons
**Color scheme (only the ones with the most votes are presented)**							
	Color scheme 7—pink, blue, green, yellow, and orange	7		—^a^		—	
	Color scheme 5—reds and grays	6		—		—	
**Animation style (only the ones with the most votes are presented)**							
	Animation 4—the pursuit of happiness	12		Felt relatable as it represented “real” people. “Felt like I could identify myself with the animations better because it used ‘real’ people.”		—	
	Animation 1—letting go of stress	10		It worked well because it did not contain that many things. It felt very calming.		The text could be presented a bit slower. It was boring. It was too fast.	
	Animation 2—changing perspective	10		“I liked that everything was quite fast because it shows how chaotic your thoughts can be.”		—	
**Expert videos**							
	Expert 3—female actress	8		She seemed to be more like a psychologist. It was nice that she also used her body while speaking. “I liked the calm background and her voice.” It was nice and simple without any disruptive elements. She seemed trustworthy and professional.		—	
	Expert 1—male professor	7		Seemed smart. “His way of talking appealed the most to me, but the books behind him didn’t.” “He seems intelligent. The books only added to his professionalism.”		“A bit ‘heavy.’ He seems like a know-it-all.” “It is way too boring, you don’t actually listen to what he says.” “The books in the background are disruptive. I can’t really focus on him because of them.” The background is ‘too noisy.’	
	Expert 2—2 young people	6		It was nice that young people presented the information as it made it feel more relatable. “It felt more age appropriate to have young people speak to me, but it also depends on what they’re talking about.”		It would be nicer to only have one person talking. It was confusing that they alternated talking. They were a bit stiff.	
**Push notifications**							
	Notification 5—“You’re well on your way. How are you today?”	12		“It feels motivating to receive praise and a reminder in one.” “It is nice that it has both a motivating text and a question.” “It is nice that something positive is included.” “It feels like a pat on the back.”		—	
	Notification 1—“It has been a while since we’ve heard from you. How are you?”	10		“It feels like a friend is asking.” “It is good.”		“Can be understood both positively and negatively.”	
	Notification 2—“We know, you’re working hard. Have you rewarded yourself?”	9		“The thought of taking care of yourself is nice.” “I like the idea of rewarding myself.”		“Can maybe be a bit demanding.”	
	Notification 3—“How has your day been?”	4		“Covers a lot.”		“Is hard to answer as it is too broad.”	
	Notification 6—“Tell us how your day has been?”	2		“Is a simple, open-ended question.”		“Seems like an order.” “It sounds a bit negative.”	
	Notification 4—“How is practicing your steps going?”	0		More specific		Not very deep “Seems like a yes/no question.” “Can seem like a reprimand.”	
**In-program feedback—ratings**							
	Rating 1—smiley faces	13		“It is easier to understand faces as opposed to e.g., colors.” The intention and meaning were clear. “The variety of options is a plus.” “It doesn’t sugar coat anything, which is nice.”		“Maybe faces can be too specific.”	
	Rating 2—weather	7		“It does make good sense to compare your mood to the weather.” “The weather gives you more room for interpretation and ascribing emotions.”		“It is a bit difficult to understand.”	
	Rating 3—waves	1		The waves did leave more room for interpretation.		“Needs more options.” “It is too unspecific. It can’t relate to it at all.” “I don’t understand it.”	
**In-program feedback—completion**							
	Feedback 1—checkmark, praise, and quote	9		It was simple and serious. “I like the combination.” “It is good and easy to understand.” “I really like the use of quotes.” “It seems the most professional and the quote is like a reward.”		—	
	Feedback 2—animated character receiving a trophy and confetti on a podium	8		“The progress-bar is nice as you can track your progress and see how much is left of the program.” “It is nice that it has no gender.” “It is cute.”		“It is a bit too over the top.” “It needs to be used consequently otherwise you’d think you weren’t doing good enough.” “It is a bit too ‘cute.’”	
	Feedback 3—animated character waving as it walks along a progress bar	3		“It is nice to have some movement in the animation.” “It is funny.” “It is good to know how far you’ve come.”		“It seems too childish.”	

^a^Not applicable; the adolescents did not have any comments on the specific prototype listed.

#### Color Schemes

Color scheme 7 received the most votes (7 votes), closely followed by color scheme 5 (6 votes).

#### Animation Style

Animation 4 received the most votes (12 votes), closely followed by animations 1 and 2 (tied; 10 votes each). The adolescents described animation 4 as the most “relatable” one. They thought that it “worked well” because it included “humans” and that they were able to “identify better” with the animation because of this.

#### Expert Videos

Votes were almost tied between the expert videos. Expert 3 received the most votes (8 votes), followed by expert 1 (7 votes) and expert 2 (6 votes). The adolescents described how expert 3 seemed more like a psychologist in the way she looked and talked. They also preferred the background and simple setup of expert 3 (ie, plain background) compared to that of expert 1 (ie, shelves with books) as it was perceived as more “calm” and not “disrupting.” However, some adolescents felt that the look and background of expert 1 added to the credibility of the expert and the information provided in the video.

#### Push Notifications

Notification 5 received the most votes (12 votes). The adolescents emphasized the motivating and acknowledging aspect of receiving praise and that it felt nice to combine praise and a question in contrast to just a question (ie, “You’re well on your way. How are you today?”). The adolescents also described how the tone of the text felt more like “a friend” and “supportive.” Notification 1 also received a large number of votes (10 votes; “It has been a while since we’ve heard from you. How are you?”). It was also described as feeling “like a friend.” However, most adolescents also pointed to the potential “double meaning” as they thought that it could be “interpreted both as positive and negative.” The same was the case for notification 2 (9 votes), which was described as both “pleasant” because of “the thought of caring for yourself” and “commanding.”

Notifications 4 and 6 received the least votes and were described as “negative” and feeling like “being told what do to” (eg, “Tell us how your day has been?”). Notification 3 was perceived as too hard to answer because it was too “broad.” However, some adolescents did like that it was not too specific and would “cover more.”

#### Ratings

Rating 1, an array of different smiley faces, received the most votes (13 votes). The adolescents described how it was “the easiest to understand the meaning of” and that it was “an advantage that it had more options” to describe their mood more accurately. Ratings 2 (7 votes) and 3 (1 vote) were generally perceived as “hard to interpret,” “difficult to understand,” and too “unspecific.” However, the metaphors of weather and waves could also “leave room for interpretation” and the “possibility to attribute more feelings to it” compared to the smiley faces in rating 1.

#### Completion

Feedback 1 and 2 received the most votes (9 and 8 votes, respectively). The adolescents preferred more “serious”-looking feedback and animations, such as simple checkmarks or written praise and quotes, and they wanted the animations to be the same “or else you could think that you’ve not done it well enough.” The adolescents were very fond of the combination of praise and a quote as the quote then felt like “a reward” (eg, “Good job! As Walt Disney once said, the difference between winning and losing is not quitting”). Feedback 3 (3 votes) was perceived as “too immature,” but the adolescents liked the visualization of how far they had come in the program.

#### Parental Involvement

When investigating adolescents’ help-seeking behavior in the user involvement workshop, the general response on how to talk with parents about something difficult was to “just say it as it is” as “your parents want what’s best for you anyway.” However, all groups also mentioned seeking support from others, such as another family member (eg, grandparent), a teacher, friends, the school nurse, a physician, counseling services, or the internet, despite all scenarios being centered on parental involvement.

When looking at overarching themes across all scenarios, the themes *security* and *respect, boundaries, and mutual understanding* were identified (Multimedia Appendix 3). *Security* represents the feeling of relational safety (“they only want what’s best for you”) that enables the adolescent to feel comfortable speaking to someone else. *Respect, boundaries, and mutual understanding* represents the adolescents’ need for autonomy (“she needs to establish boundaries for her parents”) and having the defining voice in how to communicate about their difficulties (“Make agreements with your parents on what they can ask about and when”).

### Phase 2: Testing Intervention Prototypes in Usability Tests

The results from the usability tests can be found in Multimedia Appendix 3. Generally, the interface of the session was perceived as intuitive and easy to navigate by the adolescents. They expressed that “it is easy to understand, what I have to do” to move on in the program. However, most of the adolescents, especially the younger participants, felt that the session was “too long” and that there was “a bit too much text” and “too many questions.” One of the youngest participants commented that she had “actually forgotten the video” she saw at the beginning of the session when she had to refer to it in an assignment at the end of the session.

The content was generally perceived as “understandable.” Both graphics and videos were generally perceived as helpful to better understand the techniques and examples presented. However, for the youngest participants, some of the content was too complex at times. One participant explained that the animations were “good but at times confusing” if too much was happening at once. She noted that “you have to look at them for a while to understand them.” Another participant commented that the graphics helped her understand the content better and that the order of presentation of content was important for her. She explained that it would be more helpful to see the graphics first and then the text-based explanation for her to better understand it (eg, seeing the feelings thermometer as a graphic and then reading about it).

### Phase 3: Feasibility Tests With End Users

The results from the interviews conducted with families in treatment during the feasibility trial can be found in Multimedia Appendix 3.

#### Satisfaction and Acceptability

The participants were generally satisfied with the program content. Both adolescents and parents emphasized graphic elements such as animations, videos, and audio files as particularly beneficial for their understanding and acquiring of the treatment techniques. Both adolescents and parents noted how the audio files with stories from past patients with anxiety disorders were “relatable” and “helped them understand” their own or their children’s anxiety. One adolescent noted that it helped him feel like he was “not the only one who has got something.”

Both adolescents and parents also liked that the introductory sessions in each of their programs included videos on how to navigate the platform and functionalities within the program. One parent noted that “there were videos for everything, so it was impossible to do anything wrong.” Another parent also noted that it was “helpful” that she “could always go back and watch it again if you needed to.”

Parents particularly liked that they, in their program, were able to try out some of the same techniques and tasks as their children but directed at their own worries (ie, cognitive restructuring or doing an exposure task). One parent explained how this made her more capable of helping her child throughout the treatment. However, for some participants, it was still difficult to understand how to do the exposure tasks. Some of the parents pointed out that they would have liked to be presented with more disorder-specific examples of *stepladders* and *step planners*.

Almost all participants, both adolescents and parents, expressed that the amount of reading was too much. In addition, some participants found the number of questions and tasks to complete in each session overwhelming. One adolescent explained that he “needed to take breaks after doing tasks to stay motivated.” One of the parents also said that the breaks within the sessions “felt like an acknowledgement and like it was okay to not complete everything in one go.”

#### Parental Involvement

Similarly to the findings of the user involvement workshops, adolescents expressed how having their own treatment program separate from their parents gave them a sense of “freedom” and “confidentiality” regarding the program. Parents, on the other hand, expressed feeling a “lack of control” or being “excluded.” However, some parents (of mainly older children) also noted that the lack of control or unawareness of the adolescent’s progress was “maybe healthy” and “fine but took a little getting used to.”

Both adolescents and parents did find it difficult to align their progress when completing separate programs. Some families noted that it was “weird” or “difficult to discuss the program content when they were not at the same session” and that it would sometimes lead to “misunderstandings.” Another family noted that they sometimes had difficulties helping their adolescent with their tasks if they had not been introduced to the techniques yet.

### Final Iteration

The first versions of the adolescent and parent programs were completed in December 2022 and evaluated in a feasibility trial from January 2023 to June 2023. Findings from the interviews conducted during the feasibility trial (described previously) were then used to update and expand existing material from the first version. The main updates made to the program were (1) editing down of material and converting text to graphics, (2) including in-session breaks to help adolescents stay motivated, and (3) aligning the adolescent and parent programs to be completed simultaneously. The second versions of the 2 programs are currently being evaluated in an RCT (ClinicalTrials.gov NCT06368557).

The adolescent and parent versions of the program content can be found in Multimedia Appendix 4.

## Discussion

### Principal Findings and Implications for Content Development

#### Overview

This study addresses a gap in the current research on developing DMHIs. First, this study provides examples of feasible methods of including end users in the initial stages of development processes. Second, the results of this study complement and expand the current knowledge on adolescent preferences with regard to DMHIs. This may ensure the development of relevant DMHIs and help establish the validity of these interventions for implementation in routine care settings.

Results from phase 1 guided the look and feel of the intervention content, such as the graphic identity (ie, colors and animations) and communication style. Generally, adolescents liked content that was relatable and age appropriate. Adolescents preferred communication that felt “like a friend” (ie, in a tone that was supportive). When discussing support and help-seeking behaviors, adolescents emphasized both themes of independence and the need for relational security. In addition, they pointed out how support may be provided not only by parents but also by others in their social circle. In phase 2, adolescents perceived the platform as intuitive and easy to navigate. Overall, the content was easy to understand but too lengthy. In phase 3, families were generally satisfied with the intervention content, but it was still too lengthy, and the 2 programs (ie, the adolescent and parent programs) needed to be better aligned to allow for adolescents and parents to progress at the same pace. Overall, feedback from end users helped identify areas for further improvement.

#### Objective 1: Preferences on Graphic Identity and Communication Style

##### Overview

Findings from the user involvement workshop helped clarify adolescent preferences regarding both graphics and text-based material. The prototypes with the most votes guided the development of all content types. Thus, it was important to investigate this early in the development process while still outlining the program.

##### Graphic Identity

The color schemes and animation styles with the most votes were used when designing the project website and logo and in the design of graphic material for the intervention (Multimedia Appendix 5). Preferences on animation style and expert videos were also used when developing the graphical content of the intervention. Specifically, the somewhat equal number of votes regarding expert videos led to the inclusion of two types of experts in the program: (1) a psychologist explaining CBT techniques and psychoeducational material and (2) a professor explaining the research on anxiety and CBT. On the basis of adolescents’ qualitative comments, we decided to keep the background simple (Multimedia Appendix 5).

##### Communication Style

The qualitative comments from the adolescent discussions of prototypes of notifications and feedback guided the writing style of the program. We used a sort of “friendly strictness,” where the program should feel like a friend supporting but also pushing them forward. One way to do this was to formulate explanations and tasks as offers instead of directive statements while still emphasizing the importance of doing the tasks (ie, “A lot of people make the mistake of believing that their thoughts are truthful and factual. However, thoughts are just mere ideas that may or may not be true. You won’t know if your thought is true unless you do a fact-check of it. So, one of the first things you should ask yourself, when you’re feeling anxious is...”). Furthermore, the adolescents’ comments also emphasized the positive impact of including quotes and praise. Thus, praise was included throughout the program at the conclusion of exercises and modules (ie, “Well done! Now you should have a bunch of ideas on how to reward yourself”). At module completion, the praise was also combined with quotes as they were perceived as rewarding by the adolescents (ie, “Congratulations! You’ve now completed Step 5. Walt Disney once said that the difference between winning and losing is most often not quitting”). We decided to include quotes as relevant in-program rewards to keep adolescents motivated to use the program.

We did not end up including push notifications in the feasibility version of the program due to technical limitations within the digital platform. Instead, they have been included in the RCT version that is currently being evaluated. Their potential impact on adherence will be investigated in that trial.

##### Relatedness

In the user involvement workshop, the topic of *being relatable* was evident in the qualitative comments across all preferred prototypes. Adolescents simply preferred content prototypes that were relatable in such a way that they could identify with them, such as using humanlike characters in animations, using appropriate metaphors, and using supportive and developmentally appropriate language. This is in line with previous research emphasizing the importance of including relatable and age-appropriate content [16,35,36,41] and, thus, including adolescents in the design process to clarify which types of content are seen as relevant.

This finding guided the writing of the intervention manuscript and development of graphic material. For example, we included recurring animated characters, iGuides (Multimedia Appendix 5), that guided the adolescents through the intervention content using a storytelling approach in a cartoonlike format. The purpose was to offer a more personal and age-appropriate dissemination of CBT concepts and techniques presented in the program. We also conducted interviews with past patients whose stories were then audio recorded by actors to provide the adolescents with a feeling of recognition and acknowledgment. These stories were included as part of psychoeducation (ie, examples of how it feels to have anxiety), cognitive restructuring (ie, examples of helpful thoughts), and exposure tasks (ie, examples of goals and use of the technique) and as motivational speeches (ie, how it was to receive treatment and overcome anxiety). In addition, we included “help a friend” tasks in which the adolescent had to help a friend (ie, a fictional case) using the presented CBT techniques to further the feeling of relatedness and mastery.

The theme of being “relatable” was also present in the interviews conducted with families from the feasibility trial in phase 3. In this case, families noted that the stories from past patients had helped them feel less alone (“I’m not the only one who has got something”) and helped them understand their situation better. Thus, relatedness and relatability may be key themes when developing engaging internet-based interventions.

#### Objective 2: Help-Seeking Behavior

Insights on adolescent help-seeking behavior helped clarify how adolescents would prefer to be supported by parents and caregivers.

In the user involvement workshop, the overarching themes present in the adolescents’ discussion of support and help-seeking behavior tapped into the duality of taking charge on their own (eg, by “establishing boundaries,” “making agreements,” and being “serious”) and the need for relational security with others in this process (eg, by “just telling them” and “talking to someone you trust”). In addition, many adolescents also qualitatively emphasized how it could be helpful to speak to someone other than their parents (eg, a friend, a teacher, or another family member).

This duality may reflect how adolescents themselves initiate and engage in scaffolding efforts as learners. Scaffolding refers to the process “that enables a child or novice to solve a task or achieve a goal that would be beyond his unassisted efforts” [42]. Thus, it refers to the process of assisting a person to move through the zone of proximal development by “controlling those elements of the task that are initially beyond the learner’s capability, thus permitting him to concentrate upon and complete only those elements that are within his range of competence” [42]. In this case, adolescents may themselves initiate this process by seeking out relevant “assistants” to support them in using the intervention and acquiring the skills and knowledge presented in the intervention.

In addition, there is some evidence suggesting that social support may be more important in ICBT treatments in which the level of therapist support is low compared to traditional CBT. In a predictor analysis by Spence et al [37], social support from family, friends, or a special person significantly predicted higher levels of program adherence for adolescents completing the BRAVE-online self-help program. However, only family support predicted treatment outcome. This may underline the importance of parental support to increase treatment adherence in digital formats.

However, in a predictor analysis by Stjerneklar et al [43], the time spent by parents helping their adolescents complete treatment did not significantly predict treatment outcome. In this study, the level of therapist support was high (weekly phone calls set to a duration of approximately 20 minutes) compared to that in the study by Spence et al [37] (no support). Thus, the role of parental support may only be important in interventions with no or low levels of therapist support.

In broader terms, these themes may also reflect how adolescence as a developmental stage is a time when liberation from parents and the need for autonomy become more apparent. Turning to peers instead of parents for support may not be unusual for this age group, and thus, the program content should be inclusive of this. In addition, increasing autonomy may call for a communicative approach that emphasizes the adolescents’ agency [44]. This meant that, instead of focusing solely on using parents for support during the ICBT treatment, adolescents were also prompted to create a list of their “support network” in the program’s first session, identifying all people who would be able to support them and specifying how they would like to be supported by them.

In the feasibility trial conducted in phase 3 of the development process, autonomy and support were also themes that were discussed by the families. Adolescents generally enjoyed the autonomy related to having separate programs, whereas for parents, this was associated with mixed responses. Generally, parents of older adolescents were more positive toward their children working in the program on their own. This is in line with findings from a co-design study by Ludlow et al [45] with caregivers of young people aged 7 to 17 years. They described how the desired level of caregiver involvement depended on the young person’s age, where involvement may be lower with older adolescents. Thus, allowing for different levels of caregiver involvement depending on the age of the adolescent could allow for more flexible and personalized use of the intervention.

#### Objective 3: Navigating the Platform and Content

The usability tests provided practical information on how end users would navigate the platform and content. On the basis of this, the intervention material was edited down significantly, and most text bodies were converted into graphics or short videos. On the basis of feedback from the youngest participants (aged 12 years), some of the existing graphics were also simplified and slowed down. Where possible, existing videos were edited down or split into sections to not last more than 2 to 3 minutes each. These findings were then used to guide the design of subsequent graphic elements.

#### Objective 4: First Impressions From Families in Treatment

Generally, families were satisfied with the program content and platform. Families emphasized the graphic elements of the content, such as animations, videos, and audio files, as particularly beneficial. On the basis of this, some text bodies were converted into graphics and videos, and other text-based elements such as checklists (eg, lists with examples of symptoms, behaviors, and rewards) were edited down. One session (on realistic thinking) was also split into 2 subsessions on identifying and working with anxious thoughts and practicing detective thinking.

However, some families did find it difficult to align their progress when completing separate programs. This led to 2 major revisions to the parent program related to enhancing the joint use of the 2 programs. First, guidelines on how to appropriately use the program were included in the introductory session. This included guidelines on how often to work with the program, how often to do exercises outside of the program, and tips on how to stay consistent with the help of parents, all with the purpose of better aligning the adolescent and parent progress when working separately. Second, the parent program was also restructured from 7 sessions to 10 sessions to better reflect the course of the adolescent program. Coping strategies presented in the adolescent program (in the “toolbox” session) were included in short in a “toolbox” session for the parents. In addition, the intended purpose and rationale of each task were elaborated on further in both the adolescent and parent programs to help families establish a mutual understanding of the program content.

Despite efforts to keep program content short, it was still too extensive, and the amount of reading was too much. In addition to converting text bodies to graphics and editing down the material, more in-session breaks were included, encouraging adolescents to take time to implement the strategies and then return to the platform.

Thus, findings from the feasibility interviews led to substantial revisions of the program, all related to enhancing its use. However, little is known about the role of including user-centered practices to enhance engagement with DMHIs. Some research conducted with adult populations indicates that using user-centered practices may help increase engagement [46,47], but more research is generally needed.

### Limitations

This study has some limitations related to the representativeness of the samples and the extent of the involvement of end users in the design processes that may be of importance when interpreting the results.

Not all samples in this study may be representative of the actual end users, who are children with clinical anxiety. However, this argument assumes that there would be a difference in preferences between adolescents with and without anxiety, but perhaps it is feasible to first include adolescents as experts on *being young* as opposed to (but not exclusive of) *being anxious* when we want to gain knowledge on adolescent preferences in general.

Furthermore, demographic data on ethnicity and socioeconomic status were not collected for participants in phases 1 and 2, and it is unclear whether our sample was demographically representative. Future research could focus on collecting these data and recruiting participants from different locations (ie, schools in different areas, counseling services, and mental health services) to enhance diversity.

In addition, only 4 adolescents participated in the usability tests in phase 2. Although representing both the youngest and oldest adolescents in the intended age group (aged 12 and 16 years, respectively), the small number of participants limits the external validity of the results. It may be that the results obtained from the usability tests are specific to the platform and content viewed in this study. In the future, conducting usability tests online or “on-site” in places where adolescents regularly spend time (ie, in class or at youth centers) could aid the recruitment of more adolescents and increase sample size.

In this study, we emphasized the importance of systematically including adolescents as end users of an intervention. However, parents and clinicians may equally represent end users of this intervention and constitute valuable sources of information in a design and implementation context. We did include clinical psychologists working in routine care settings as part of the expert group to qualify the program manuscript but did not systematically collect data from them. This poses a limitation of this study, and it would be beneficial in the future to include all intended end users in the development processes.

It may also be that adolescents could have been involved more in the design of the intervention and at an earlier stage. In a study by Ludlow et al [16], the research team used co-design methods to understand preferences and perceptions of the design, functions, and engagement features within a new digital mental health platform. The aforementioned study used generative toolkits to facilitate the co-design processes through production of artifacts. These artifacts (and output from previous design phases) were then used to qualify the design of their new digital mental health platform for young people. This type of end-user involvement allows young people to hold a central position as experts at very early stages of the development process. However, it may also be extensive and require a lot of time and resources that may not be available.

When comparing this study to the one by Ludlow et al [16], it becomes apparent that, despite using user-centered methods, researchers have the power to define *what* end-user perspectives to include and *how* to investigate them. There may not be a way to solve this paradox, but it underlines how important it is to be aware of one’s own impact as a researcher when engaging in these practices.

### Conclusions

DMHIs have the potential to overcome treatment barriers such as stigma and costs and increase access to effective treatment at an early stage in symptom development. However, most DMHIs suffer from low treatment adherence. It has been proposed that the lack of end-user involvement in the development of intervention content and platforms may be of particular importance when interpreting the low adherence to DMHIs. In this study, we used user involvement practices at different stages of development to investigate adolescent and parent preferences on specific design components. Information from these practices was implemented when developing intervention content. This study emphasizes the importance of understanding the design process as iterative, where content prototypes are subjected to multiple phases of evaluation. This ensures the continued alignment of the intervention with end-user needs and may help establish the validity of the intervention for implementation in routine care settings. Future research may use user involvement practices such as those described in this paper to understand how to individualize intervention content and patient courses. In addition, user involvement practices could be used to investigate adolescent preferences on specific treatment components (eg, relaxation techniques and behavioral experiments) and the order in which they are presented (ie, in a specific order or as optional components).

## Appendix

Multimedia Appendix 1 Guidance for Reporting Involvement of Patients and the Public checklist in long form.

Multimedia Appendix 2 Interview guides.

Multimedia Appendix 3 Results from the usability and feasibility tests.

Multimedia Appendix 4 Overview of the content of the adolescent and parent program versions used when conducting the feasibility trial (version 1) and the randomized controlled trial (version 2).

Multimedia Appendix 5 CoolMinds intervention content.

## Abbreviations

CBT: cognitive behavioral therapy
DMHI: digital mental health intervention
ICBT: internet-based cognitive behavioral therapy
RCT: randomized controlled trial
REDCap: Research Electronic Data Capture

## Data Availability

The data used in this manuscript contain sensitive and potentially identifiable information regarding both children and caregivers, and all participants were guaranteed full anonymity. As such, and in line with Danish Data Protection Agency regulations, the data used in this study are not publicly available. Unidentifiable data excerpts can be made available upon request to the corresponding author (nikita@psy.au.dk). Further data access requests may be sent to the Technology Transfer Office at Aarhus University (tto@au.dk).
