# Supplementary material for: Developing an Internet-Based Cognitive Behavioral Therapy Intervention for Adolescents With Anxiety Disorders: Design, Usability, and Initial Evaluation of the CoolMinds Intervention
Source: JMIR Form Res. 2025 Apr 8;9:e66966. doi: 10.2196/66966 (PMC12015348; doi:10.2196/66966)
Supplement: Multimedia Appendix 2 [file formative_v9i1e66966_app2.docx]

## Multimedia Appendix 2: Supporting information.

## M1. Interview guide for usability tests

*Note:* Questions were adapted based on the age of the participant. Questions in cursive are examples of important follow-up questions based on the participant’s answer.

Questions related to user experience

1. What are your thoughts about the platform?

2. How was it to navigate the platform?

3. What or which functionalities worked well in the app?

4. What or which functionalities worked poorly in the app?

5. How would you feel about receiving treatment for anxiety in this way?

6. How would you prefer to receive treatment? *App/computer/in person? Pros/cons for each type?*

Questions related to intervention content

1. How was the dissemination of the content? *Easy/difficult/adequate to understand? When was it too easy/difficult?*

2. What parts of the content were particularly helpful to understand the treatment?

3. How was to ratio between text and graphics? *Was is too much/too little/adequate?*

4. How did you experience the duration of the session? *Too long/short/adequate? Were there times where it was difficult for you to complete the session?*

Questions related to parental involvement

1. How do you feel about having your own program that is separate from your parents’? *Pros/cons/worries?*

2. If your parents were to help you complete the treatment program, how would you like them to help you?

## M2. Interview guide for feasibility tests

*Note:* Only questions relevant in relation to the family’s progress in the program were administered, i.e., if the family had not completed the program we did not ask about their experience with the ending of the program.

| **Topic** | **Questions** |
| --- | --- |
| *General questions on intervention content* | 1. What parts of the content were particularly helpful/difficult to understand the treatment? 2. What parts of the content were particularly helpful/difficult to keep up the motivation throughout the treatment? 3. How long did you approximately spend on completing each session so far? 4. How much time have you approximately spent each week on working with the program? 5. How do you use the program? *Do you return to previous sessions?* 6. How did you perceive the ending of the program? *What elements made it feel safe/unsafe to end the program?* |
| *Text-based elements* | 1. Did you understand the content presented in the program? 2. How do you perceive the language used in the program? |
| *Graphic material* | 1. Where did it work particularly well/poorly? (movements in the graphics, amount of text in graphics) 2. Are there any places with too many/few graphics? 3. What did you think of the iGuides? *When did the use of them work well/poorly?* |
| *Videos* | 1. What do you think of the videos in the program? *What worked well/poorly, e.g., quality (sound/picture), content (understanding), persons (relatable)?* |
| *Audio* | 1. What did you think of the audio tracks in the program? *What worked well/poorly, e.g., quality, relatability?* |
| *Parental involvement* | 1. How do you feel about completing separate program as adolescent and parents? |
| *General questions on platform use* | 1. What device do you use to access the program (phone, tablet, computer) 2. How was it to navigate the platform on your device? 3. Did you experience any difficulties using the app/computer, such as technical difficulties? 4. Did you use the chat module? What do you think of it? |
