# Supplementary material for: Developing an Internet-Based Cognitive Behavioral Therapy Intervention for Adolescents With Anxiety Disorders: Design, Usability, and Initial Evaluation of the CoolMinds Intervention
Source: JMIR Form Res. 2025 Apr 8;9:e66966. doi: 10.2196/66966 (PMC12015348; doi:10.2196/66966)
Supplement: Multimedia Appendix 3 [file formative_v9i1e66966_app3.docx]

# Multimedia Appendix 3. Results from usability and feasibility tests

**Table 1.** Themes of support derived from the user-involvement workshop discussions.

| *Theme 1* | *Security* | “Your parents only want what’s best for you.”  “Tell your parents, but also emphasize how you don’t want to disappoint them.”  “They all just want what’s best for you.”  “If you need to, you could have someone help you talk to them.”  “Talk to an adult you trust.” |
| --- | --- | --- |
| *Theme 2* | *Respect, boundaries, and mutual understanding* | “She needs her space, and her parents need to respect that.”  “She needs to establish boundaries for her parents.”  “It is important to accept boundaries and to listen to each other.”  “You need to try to understand each other and take it seriously.”  “Your parents should listen and ask elaborative questions.”  “Just tell your parents about it, they will respect you.”  “Sit your parents down in a way where they know it’s serious.”  “Speak to them in a serious manner.”  “Make agreements with your parents on what they can ask about and when.” |

**Table 2.** Categorization of notes and recordings from usability tests.

| **Description** | **Usability test 1** | **Usability test 2** | **Usability test 3** | **Usability test 4** |
| --- | --- | --- | --- | --- |
| **General comments** | It takes 60-65 minutes to complete the entire module.  The module was very long, and it could benefit from including more breaks. | It took 45 minutes to complete the module without typing answers.  The green bar at the top of all pages was nice so you could track your progress.  The difficulty of the language was adequate, and the intentions seemed clear.  The participant would have preferred animated explanations instead of text.  There would be too many exercises if the participant was to fill them in. The module was also generally too long. | No comments. | The participant thought that there was a good mix of teaching techniques included which helped her focus.  The length of the text was okay, and you could easily understand the language.  The module was too long.  The participant expressed that she would prefer this type of treatment to face-to-face therapy. |
| **Comments on interface** | The “open section”-button disappears when scrolling down a page. | No comments. | No comments. | It was difficult to find the “go back”-button when going back and forth between pages. |
| **Page 1**  *Introduction* | Adequate amount of text in speech bubbles. It is nice that the graphic elements include movement.  The explanation in the video (with the psychologist) makes good sense but it is too long. You get the point relatively quickly. | The graphics are nice, and the text and video are easy to understand. | Easy to understand.  Relatable examples.  The video (with the psychologist) is a bit long. | The introduction works well to frame the module.  The video (with the psychologist) was too long, and it was difficult to concentrate at the end of it. However, the explanation made sense and made it ‘worth it’. |
| **Page 2**  *The tripart model of anxiety* | No comments. | The movement in the animated examples are too confusing and fast. | The animated examples make sense, but you have to look at them for a while to understand them. There are just too many things happening at once. | The animated example was nice but there is a spelling error. |
| **Page 3**  *My feelings depend on my thoughts* | No comments. | The history is good and easy to understand. The animations complement the history well. | The history illustrates different ways of thinking very well. You understand this point better when it is a long story using animations instead of text. | The concluding animated model summarizes the overarching points nicely. |
| **Page 4**  *Exercise 1 on the tripart anxiety model* | It was difficult to understand what was meant with the question: “What do you think she does or does not do?”  The participant couldn’t remember the examples (presented earlier) that were to be used in the exercise. | The exercise was easy to understand, and the participant did remember the examples to be used.  The participant was also able to come up with a relevant example from their own life to be used in the last part of the exercise. | The examples to be used were too far back. And because you’d been presented with different examples it was unclear which one was to be used.  It is easy to understand what is to be done in the exercise. | It was difficult to understand the question: “How do you think she feels this in her body?”. The participant did not know if she was to look at bodily sensations or feelings. |
| **Page 5**  *Understanding thoughts and feelings* | No comments. | The animation refers to a “barometer” while the text refers to a “thermometer”.  The participant is confused by the illustration of the thermometer, as she doesn’t if she’s supposed to do anything with it or not. | There was a bit much text on the page.  The explanation in the text made sense when you’d seen the illustration further down. Until then it was a bit confusing. | Some of the language reminded her of spoken language, which she felt contrasted with the writing style used up until then. |
| **Page 6**  *Exercise 2 on feelings and the bodily reactions* | No comments. | No comments. | There is very little text on the page. It is difficult to understand what this page is meant to do, as the exercise is presented but is to be done on the next page. | No comments. |
| **Page 7**  *What happens in my body when I experience difficult emotions?* | There are too many questions, and they are difficult and require a lot of time to answer. | The exercise is difficult to understand, and it requires that you read it a few times. The participant was however able to answer all questions presented in the exercise.  Some of the feelings presented in the exercise aren’t that relatable, and some feelings are difficult to understand: “*I don’t know how it would be to feel this*.” | It is nice that you’re asked about other feelings than anxiety. | Some of the feelings were difficult to understand, as it was hard to identify how they would feel. Some of the feelings were also too alike such as happy and excited. |
| **Page 8**  *Exercise 3 on measuring feelings* | The participant got confused as she’d forgotten how the thermometer worked. She would have liked the short explanation of the scale (0-10) to be present at each exercise. | It is easy to control the slider of the thermometer. The examples are relatable, and it is easy to identify feelings associated with each situation.  One of the examples (student council) was not very cool. | Very relatable examples. | Some of the examples (e.g., failing a test) were very relatable while others (grandmas’ birthday) were harder to answer because it was difficult to identify the feelings involved.  It is not cool to be part of the student council so it would not be associated with positive feelings. |
| **Page 9**  *BREAK* | No comments. | The participant would like to just move on. | No comments. | No comments. |
| **Page 10**  *Becoming aware of your thoughts* | No comments. | The animations were good. | No comments. | A specific sentence confused her. |
| **Page 11**  *Ways of thinking* | The explanation in the video (with the psychologist) is easy to understand. | The video (with the psychologists) provides good examples that are easy to understand. Specifically, the complementary illustrations in the video makes it easier to understand. | No comments. | The video (with the psychologist) is good and follows along with the examples nicely. |
| **Page 12**  *Anxious thinking* | There is an adequate number of options in the list. | There is a spelling error in the exercise.  The different ways of thinking presented were easy to understand and identify with. She thought it was a fun exercise because you didn’t have to write anything yourself. | No comments. | The list of examples was relatable and had an adequate number of options. The participant was able to identify her own ways of thinking. |
| **Page 13**  *Negative ways of thinking* | No comments. | The text is easy to understand, but it is too long. | No comments. | The participant didn’t understand what it meant to be “*stuck in your own thoughts*.” |
| **Page 14**  *New ways of thinking* | No comments. | The introduction text is mentioned as having a ‘good length’. But it would be better to present in a video.  The participant liked that the movements were slower in these animations. She generally thinks that the animations on this page are cool. | No comments. | The animations on this page are good and helpful. |
| **Page 15**  *Exercise 4 on identifying helpful thoughts* | There is an error in the exercise text and the explanation where two different words are used about the same thing. | The introduction text was easy to understand, and the examples were good.  One of the examples (playing soccer) was difficult to imagine. It was difficult to figure out what to answer here. The other example (a school exam) was more relatable. | No comments. | This exercise was very informative. |
| **Page 16**  *Exercise 5 on helping a friend* | No comments. | The participant liked that the exercise was introduced by an iGuide (character). She thought it was a lot better than the text introductions.  The exercise was easy to understand and felt relatable. | No comments. | It would be nice to have more examples related to oneself, so you’d have to use the techniques on your own thoughts. |
| **Page 17**  *Exercise 6 on identifying anxious thoughts in everyday situations* | No comments. | The exercise was easy to understand but some of the questions would require a lot of mental energy to answer. The participant did feel like she would be able to answer the questions, if she maybe had more time.  It was nice that the text fields included examples to be inspired by. | No comments. | The participant liked the idea of giving yourself advice “*as a friend*”. |
| **Page 18**  *Conclusion of the module* | No comments. | The participants liked the animations and praise from the iGuides.  The quiz was nice but some of the questions were hard to answer. | No comments. | The quiz was nice and had you reflect on the material. |

**Table 3.** Categorization of interview data collected during the feasibility trial.

|  | **Pros** | **Cons** |
| --- | --- | --- |
| **Introductory videos** | The introductory videos “were good … how to navigate [the platform, ed.]”  “*There were videos for everything, so it was impossible to do anything wrong*”.  “*You could always go back and watch it again if you needed to*”. |  |
| **Text-based elements** | “It is easy to understand.”  “It is nice that you have some facts.”  “The ratio between text and graphics was fine … not too much or too little of any of them.” | “There is quite a lot to read”  “Some of the sentences were very long.” |
| **Quizzes** | “I quite liked all the questions for us as parents. Like when you get the introduction to anxiety and the background and how I occurs, that you’re then asked about it.” |  |
| **Animations** | “There’s not been too many. There’s been too few animations.”  “… you could read them because there wasn’t too much text in the bubbles.”  “I really liked the comic strips [graphics, ed.]”  “The animations really helped me understand what was explained in the text.” |  |
| **Videos** | “The videos worked the best when explaining things because there was so much to read”  “The videos were not too long”  “If there were more videos I would probably get more out of the program”  “And that a professional tells you about the different anxiety types. What happens with the brain and such … why it occurs. I liked that.”  “I really liked the videos with the psychologist.” |  |
| **Audio files** | “… that I’m not the only who has got something.” |  |
| **Presentation of CBT techniques** | “… but we did understand the rationale [of doing the tasks themselves as parents, ed.]. So we helped each other come up with ideas on what to write.”  “[Doing the tasks as a parent, ed.] made me more capable of helping her.”  “I really like the program.” | “[The exposure tasks, ed.] were confusing. They should be called something else. I didn’t understand what I was to do first. And I became scared of making mistakes.”  “It was weird to do the stepladders for yourself because it was difficult to choose something to work on.”  “I didn’t understand the stepladders. I had to search up a video of the professor [from the expert videos, ed.] who explained how It worked. Maybe more specific examples of how a complete stepladder should look would have helped me.”  “I needed to take breaks after doing tasks to stay motivated.” |
| **Parental involvement** | “I just really like it. I like to have my own program [as an adolescent, ed.].”  “But I have to just leave it to him, that he has got it under control.”  “She only has to discuss it with me if she wants to.”  “I can keep some things to myself that I don’t feel like sharing yet.”  “It [having my own program as an adolescent, ed.] gave me a sense of freedom and confidentiality [with the program, ed.].”  “… fine but took a little getting used to [as a parent, ed.].”  “It is difficult… but maybe it’s healthy enough.” | “It was a bit weird in the beginning because I had no feel for what my child was presented with.”  “It may be difficult to work separately in a family where the adolescent isn’t that independent.”  “It was a bit weird that we aren’t at the same place in the program, because maybe I will ask for help with something and then my mom hasn’t got to that point yet.”  “It is challenging.”  “I [parent, ed.] just have to trust that he’s working well on his own and he’ll ask for help if needed.”  “It was sometimes difficult to discuss the program content when we weren’t at the same session.” |
| **Breaks** | “I would have liked to know how far I was during the session.”  The breaks “felt like an acknowledgement and like it was okay to not complete everything in one go.” | “I needed to take breaks after doing tasks to stay motivated.” |

**Table 3.** Overview of the content of the adolescent program versions used when conducting the feasibility trial (version 1) and the randomized controlled trial (version 2).

| Module | Version 1 | | Version 2 | |
| --- | --- | --- | --- | --- |
|  | Session | Content | Session | Content |
|  | | | | |
| 1 | Introduction to the platform and program | - Information about navigating the platform - Information about the treatment setup (eg, therapist contact, questionnaires, and a review of the session) - Questions about the adolescent (eg, “do you have any pets?” and “what do you dream of doing in the future?”) - Tips on how to involve and talk to people in their social network about something difficult | Introduction to the platform and program | - Information about navigating the platform - Information about the treatment setup (eg, therapist contact, questionnaires, and a review of the session) - Questions about the adolescent (eg, “do you have any pets?” and “what do you dream of doing in the future?”) - Tips on how to involve and talk to people in their social network about something difficult |
| 2 | Psychoeducation—on specific anxiety disorders | - Identifying anxiety symptoms and intensity, anxious behaviors, and how this affects their everyday life - A self-administered test to figure out which type of anxiety would be beneficial to work with - Disorder-specific psychoeducation on social phobia, generalized anxiety disorder, separation anxiety disorder, specific phobias, panic disorder, and OCD^a^ - A quiz on matching statements and anxiety disorders (eg, “It feels like all of my thoughts are worries”—matched with generalized anxiety disorder) | Psychoeducation—on specific anxiety disorders | - Subsession 1: identifying anxiety symptoms and intensity, anxious behaviors, and how this affects their everyday life; a self-administered test to figure out which type of anxiety would be beneficial to work with; and in-session break - Subsession 2: disorder-specific psychoeducation on social phobia, generalized anxiety disorder, separation anxiety disorder, specific phobias, panic disorder, and OCD and a quiz on matching statements and anxiety disorders (eg, “It feels like all of my thoughts are worries”—matched with generalized anxiety disorder) |
| 3 | Psychoeducation—on anxiety in general | - Information on common anxiety symptoms and why anxiety occurs (eg, anxiety curve) - Introduction to the cognitive triangle - Identifying situations in which anxiety occurs using behavioral chain analysis - Introduction to using the feelings thermometer to measure anxiety - Short introduction to thought patterns and how to identify negative or irrational thoughts and create alternative thoughts - Quiz on anxiety terminology (eg, “what is an automatic thought?”) | Psychoeducation—on anxiety in general | - Subsession 1: information on common anxiety symptoms and why anxiety occurs (eg, anxiety curve), introduction to the cognitive triangle, identifying situations in which anxiety occurs using behavioral chain analysis, introduction to using the feelings thermometer to measure anxiety, and in-session break - Subsession 2: introduction to thought patterns and how to identify negative or irrational thoughts and create alternative thoughts and quiz on anxiety terminology (eg, “what is an automatic thought?”) |
| 4 | Realistic thinking (detective thinking) | - Introduction to common cognitive biases - Cognitive restructuring and detective thinking tasks - Quiz on cognitive biases and detective thinking | Realistic thinking (detective thinking) | - Subsession 1: introduction to common cognitive biases and in-session break - Subsession 2: cognitive restructuring and detective thinking tasks and quiz on cognitive biases and detective thinking |
| 5 | Goals and rewards | - Creating SMART^b^ goals - Contingency management | Goals and rewards | - Creating SMART goals - Contingency management |
| 6 | Exposure | - Safety behaviors - Anxiety curve (in relation to exposure therapy) - Gradual exposure using stepladders and step planners - Disorder-specific examples of stepladders for social phobia, generalized anxiety disorder, separation anxiety disorder, specific phobias, panic disorder, and OCD - Creating their own exposure hierarchy (stepladders) based on their goals - Quiz on safety and avoidance behaviors and stepladders | Exposure | - Subsession 1: safety behaviors, anxiety curve (in relation to exposure therapy), and in-session break - Subsession 2: gradual exposure using stepladders and step planners; disorder-specific examples of stepladders for social phobia, generalized anxiety disorder, separation anxiety disorder, specific phobias, panic disorder, and OCD; creating their own exposure hierarchy (stepladders) based on their goals; and quiz on safety and avoidance behaviors and stepladders |
| 7 | Experiments | - Behavioral experiments - Stand-alone experiments | Disorder-specific exposure task session | - Repeatable disorder-specific sessions with exposure tasks for social phobia, generalized anxiety disorder, separation anxiety disorder, specific phobias, panic disorder, and OCD |
| 8 | Toolbox | - Coping strategies such as breathing exercises (eg, calm breathing), attention control exercises, assertiveness training, urge surfing, worry-time exercises, and problem-solving techniques | Experiments | - Behavioral experiments - Stand-alone experiments |
| 9 | Relapse prevention | - Skill overview and maintenance - Self-assessment of current or near-future challenges - Tips on where and how to seek help | Toolbox | - Coping strategies such as breathing exercises (eg, calm breathing), attention control exercises, assertiveness training, urge surfing, worry-time exercises, and problem-solving techniques |
| 10 | Completion | - Diploma for completion - Tips on how to stay motivated and continue working | Relapse prevention | - Skill overview and maintenance - Self-assessment of current or near-future challenges - Tips on where and how to seek help |
| 11 | —^c^ | — | Completion | - Diploma for completion - Tips on how to stay motivated and continue working |
| 12 | — | — | Booster | - Refreshing exposure techniques and relapse prevention plans |

^a^OCD: obsessive-compulsive disorder.

^b^SMART: specific, measurable, achievable, relevant, and time bound.

^c^No content.

**Table 4.** Overview of the parent program versions used when conducting the feasibility trial (version 1) and the randomized controlled trial (version 2).

| Module | Version 1 | | Version 2 | |
| --- | --- | --- | --- | --- |
|  | Session | Content | Session | Content |
|  | | | | |
| 1 | Introduction to the platform and program | - Information about navigating the platform - Information about the treatment setup (eg, therapist contact, questionnaires, and a review of the session) - Questions about the adolescent (eg, “do you have any pets?” and “what do you dream of doing in the future?”) - Tips on how to involve and talk to people in their social network about something difficult | Introduction to the platform and program | - Information about navigating the platform - Information about the treatment setup (eg, therapist contact, questionnaires, and a review of the session) - Questions about the adolescent (eg, “do you have any pets?” and “what do you dream of doing in the future?”) - Tips on how to involve and talk to people in their social network about something difficult |
| 2 | Psychoeducation | - Information on common anxiety symptoms and why anxiety occurs (eg, anxiety curve) - Introduction to the cognitive triangle - Disorder-specific psychoeducation on social phobia, generalized anxiety disorder, separation anxiety disorder, specific phobias, panic disorder, and OCD^a^ - Introduction to safety and avoidance behaviors | Psychoeducation | - Information on common anxiety symptoms and why anxiety occurs (eg, anxiety curve) - Introduction to the cognitive triangle - Disorder-specific psychoeducation on social phobia, generalized anxiety disorder, separation anxiety disorder, specific phobias, panic disorder, and OCD - Introduction to safety and avoidance behaviors |
| 3 | Parent behavior | - Introduction to supportive behaviors such as active listening, empathy, patience, respect, and acceptance - Tips on how to regulate their own emotions - Identifying their own anxiety-maintaining behaviors (eg, reassurance, avoidance, and social learning) | Parent behavior | - Introduction to supportive behaviors such as active listening, empathy, patience, respect, and acceptance - Tips on how to regulate their own emotions - Identifying their own anxiety-maintaining behaviors (eg, reassurance, avoidance, and social learning) |
| 4 | Do it yourself | - Introduction to key techniques and tasks that the adolescents are working with, such as identifying thoughts and feelings, cognitive restructuring (detective thinking), cognitive biases, SMART^b^ goals, contingency management, and gradual exposure | Anxiety treatment—realistic thinking | - Introduction to common cognitive biases - Identifying thoughts and feelings - Cognitive restructuring - Do-it-yourself exercises on measuring feelings and detective thinking |
| 5 | School | - Information on how to involve the school if necessary and on how to work with anxiety in the school setting - Educational material for school personnel, such as teachers or pedagogues, including guidelines on how to handle bullying | Anxiety treatment—goals and rewards | - Creating SMART goals - Contingency management - Do-it-yourself exercise on using rewards |
| 6 | Relapse prevention | - Continuous practice of supportive behaviors - Identifying relapse behaviors and negative coping strategies | Anxiety treatment—exposure | - Safety behaviors - Gradual exposure using stepladders and step planners - Examples of complete stepladders - Do-it-yourself exercise on creating a stepladder |
| 7 | Completion | - Information on how and where to seek support or additional help | Toolbox | - Coping strategies such as breathing exercises (eg, calm breathing), attention control exercises, assertiveness training, urge surfing, worry-time exercises, and problem-solving techniques |
| 8 | —^c^ | — | School | - Information on how to involve the school if necessary and on how to work with anxiety in the school setting - Educational material for school personnel, such as teachers or pedagogues, including guidelines on how to handle bullying |
| 9 | — | — | Relapse prevention | - Continuous practice of supportive behaviors - Identifying relapse behaviors and negative coping strategies |
| 10 | — | — | Completion | - Information on how and where to seek support or additional help |
| 11 | — | — | Booster | — |

^a^OCD: obsessive-compulsive disorder.

^b^SMART: specific, measurable, achievable, relevant, and time bound.

^c^No content.
