# Supplementary material for: Developing an Internet-Based Cognitive Behavioral Therapy Intervention for Adolescents With Anxiety Disorders: Design, Usability, and Initial Evaluation of the CoolMinds Intervention
Source: JMIR Form Res. 2025 Apr 8;9:e66966. doi: 10.2196/66966 (PMC12015348; doi:10.2196/66966)
Supplement: Multimedia Appendix 4 [file formative_v9i1e66966_app4.docx]

# Multimedia Appendix 4. Overview of the content of the adolescent and parent program versions.

**Table S1.** Overview of the content of the adolescent program versions used when conducting the feasibility trial (version 1) and the randomized controlled trial (version 2).

| Module | Version 1 | | Version 2 | |
| --- | --- | --- | --- | --- |
|  | Session | Content | Session | Content |
|  | | | | |
| 1 | Introduction to the platform and program | - Information about navigating the platform - Information about the treatment setup (eg, therapist contact, questionnaires, and a review of the session) - Questions about the adolescent (eg, “do you have any pets?” and “what do you dream of doing in the future?”) - Tips on how to involve and talk to people in their social network about something difficult | Introduction to the platform and program | - Information about navigating the platform - Information about the treatment setup (eg, therapist contact, questionnaires, and a review of the session) - Questions about the adolescent (eg, “do you have any pets?” and “what do you dream of doing in the future?”) - Tips on how to involve and talk to people in their social network about something difficult |
| 2 | Psychoeducation—on specific anxiety disorders | - Identifying anxiety symptoms and intensity, anxious behaviors, and how this affects their everyday life - A self-administered test to figure out which type of anxiety would be beneficial to work with - Disorder-specific psychoeducation on social phobia, generalized anxiety disorder, separation anxiety disorder, specific phobias, panic disorder, and OCD^a^ - A quiz on matching statements and anxiety disorders (eg, “It feels like all of my thoughts are worries”—matched with generalized anxiety disorder) | Psychoeducation—on specific anxiety disorders | - Subsession 1: identifying anxiety symptoms and intensity, anxious behaviors, and how this affects their everyday life; a self-administered test to figure out which type of anxiety would be beneficial to work with; and in-session break - Subsession 2: disorder-specific psychoeducation on social phobia, generalized anxiety disorder, separation anxiety disorder, specific phobias, panic disorder, and OCD and a quiz on matching statements and anxiety disorders (eg, “It feels like all of my thoughts are worries”—matched with generalized anxiety disorder) |
| 3 | Psychoeducation—on anxiety in general | - Information on common anxiety symptoms and why anxiety occurs (eg, anxiety curve) - Introduction to the cognitive triangle - Identifying situations in which anxiety occurs using behavioral chain analysis - Introduction to using the feelings thermometer to measure anxiety - Short introduction to thought patterns and how to identify negative or irrational thoughts and create alternative thoughts - Quiz on anxiety terminology (eg, “what is an automatic thought?”) | Psychoeducation—on anxiety in general | - Subsession 1: information on common anxiety symptoms and why anxiety occurs (eg, anxiety curve), introduction to the cognitive triangle, identifying situations in which anxiety occurs using behavioral chain analysis, introduction to using the feelings thermometer to measure anxiety, and in-session break - Subsession 2: introduction to thought patterns and how to identify negative or irrational thoughts and create alternative thoughts and quiz on anxiety terminology (eg, “what is an automatic thought?”) |
| 4 | Realistic thinking (detective thinking) | - Introduction to common cognitive biases - Cognitive restructuring and detective thinking tasks - Quiz on cognitive biases and detective thinking | Realistic thinking (detective thinking) | - Subsession 1: introduction to common cognitive biases and in-session break - Subsession 2: cognitive restructuring and detective thinking tasks and quiz on cognitive biases and detective thinking |
| 5 | Goals and rewards | - Creating SMART^b^ goals - Contingency management | Goals and rewards | - Creating SMART goals - Contingency management |
| 6 | Exposure | - Safety behaviors - Anxiety curve (in relation to exposure therapy) - Gradual exposure using stepladders and step planners - Disorder-specific examples of stepladders for social phobia, generalized anxiety disorder, separation anxiety disorder, specific phobias, panic disorder, and OCD - Creating their own exposure hierarchy (stepladders) based on their goals - Quiz on safety and avoidance behaviors and stepladders | Exposure | - Subsession 1: safety behaviors, anxiety curve (in relation to exposure therapy), and in-session break - Subsession 2: gradual exposure using stepladders and step planners; disorder-specific examples of stepladders for social phobia, generalized anxiety disorder, separation anxiety disorder, specific phobias, panic disorder, and OCD; creating their own exposure hierarchy (stepladders) based on their goals; and quiz on safety and avoidance behaviors and stepladders |
| 7 | Experiments | - Behavioral experiments - Stand-alone experiments | Disorder-specific exposure task session | - Repeatable disorder-specific sessions with exposure tasks for social phobia, generalized anxiety disorder, separation anxiety disorder, specific phobias, panic disorder, and OCD |
| 8 | Toolbox | - Coping strategies such as breathing exercises (eg, calm breathing), attention control exercises, assertiveness training, urge surfing, worry-time exercises, and problem-solving techniques | Experiments | - Behavioral experiments - Stand-alone experiments |
| 9 | Relapse prevention | - Skill overview and maintenance - Self-assessment of current or near-future challenges - Tips on where and how to seek help | Toolbox | - Coping strategies such as breathing exercises (eg, calm breathing), attention control exercises, assertiveness training, urge surfing, worry-time exercises, and problem-solving techniques |
| 10 | Completion | - Diploma for completion - Tips on how to stay motivated and continue working | Relapse prevention | - Skill overview and maintenance - Self-assessment of current or near-future challenges - Tips on where and how to seek help |
| 11 | —^c^ | — | Completion | - Diploma for completion - Tips on how to stay motivated and continue working |
| 12 | — | — | Booster | - Refreshing exposure techniques and relapse prevention plans |

^a^OCD: obsessive-compulsive disorder.

^b^SMART: specific, measurable, achievable, relevant, and time bound.

**Table S2.** Overview of the parent program versions used when conducting the feasibility trial (version 1) and the randomized controlled trial (version 2).

| Module | Version 1 | | Version 2 | |
| --- | --- | --- | --- | --- |
|  | Session | Content | Session | Content |
|  | | | | |
| 1 | Introduction to the platform and program | - Information about navigating the platform - Information about the treatment setup (eg, therapist contact, questionnaires, and a review of the session) - Questions about the adolescent (eg, “do you have any pets?” and “what do you dream of doing in the future?”) - Tips on how to involve and talk to people in their social network about something difficult | Introduction to the platform and program | - Information about navigating the platform - Information about the treatment setup (eg, therapist contact, questionnaires, and a review of the session) - Questions about the adolescent (eg, “do you have any pets?” and “what do you dream of doing in the future?”) - Tips on how to involve and talk to people in their social network about something difficult |
| 2 | Psychoeducation | - Information on common anxiety symptoms and why anxiety occurs (eg, anxiety curve) - Introduction to the cognitive triangle - Disorder-specific psychoeducation on social phobia, generalized anxiety disorder, separation anxiety disorder, specific phobias, panic disorder, and OCD^a^ - Introduction to safety and avoidance behaviors | Psychoeducation | - Information on common anxiety symptoms and why anxiety occurs (eg, anxiety curve) - Introduction to the cognitive triangle - Disorder-specific psychoeducation on social phobia, generalized anxiety disorder, separation anxiety disorder, specific phobias, panic disorder, and OCD - Introduction to safety and avoidance behaviors |
| 3 | Parent behavior | - Introduction to supportive behaviors such as active listening, empathy, patience, respect, and acceptance - Tips on how to regulate their own emotions - Identifying their own anxiety-maintaining behaviors (eg, reassurance, avoidance, and social learning) | Parent behavior | - Introduction to supportive behaviors such as active listening, empathy, patience, respect, and acceptance - Tips on how to regulate their own emotions - Identifying their own anxiety-maintaining behaviors (eg, reassurance, avoidance, and social learning) |
| 4 | Do it yourself | - Introduction to key techniques and tasks that the adolescents are working with, such as identifying thoughts and feelings, cognitive restructuring (detective thinking), cognitive biases, SMART^b^ goals, contingency management, and gradual exposure | Anxiety treatment—realistic thinking | - Introduction to common cognitive biases - Identifying thoughts and feelings - Cognitive restructuring - Do-it-yourself exercises on measuring feelings and detective thinking |
| 5 | School | - Information on how to involve the school if necessary and on how to work with anxiety in the school setting - Educational material for school personnel, such as teachers or pedagogues, including guidelines on how to handle bullying | Anxiety treatment—goals and rewards | - Creating SMART goals - Contingency management - Do-it-yourself exercise on using rewards |
| 6 | Relapse prevention | - Continuous practice of supportive behaviors - Identifying relapse behaviors and negative coping strategies | Anxiety treatment—exposure | - Safety behaviors - Gradual exposure using stepladders and step planners - Examples of complete stepladders - Do-it-yourself exercise on creating a stepladder |
| 7 | Completion | - Information on how and where to seek support or additional help | Toolbox | - Coping strategies such as breathing exercises (eg, calm breathing), attention control exercises, assertiveness training, urge surfing, worry-time exercises, and problem-solving techniques |
| 8 | —^c^ | — | School | - Information on how to involve the school if necessary and on how to work with anxiety in the school setting - Educational material for school personnel, such as teachers or pedagogues, including guidelines on how to handle bullying |
| 9 | — | — | Relapse prevention | - Continuous practice of supportive behaviors - Identifying relapse behaviors and negative coping strategies |
| 10 | — | — | Completion | - Information on how and where to seek support or additional help |
| 11 | — | — | Booster | — |

^a^OCD: obsessive-compulsive disorder.

^b^SMART: specific, measurable, achievable, relevant, and time bound.
