# Supplementary material for: Developing an Internet-Based Cognitive Behavioral Therapy Intervention for Adolescents With Anxiety Disorders: Design, Usability, and Initial Evaluation of the CoolMinds Intervention
Source: JMIR Form Res. 2025 Apr 8;9:e66966. doi: 10.2196/66966 (PMC12015348; doi:10.2196/66966)

# Multimedia Appendix 5. CoolMinds intervention content

Multimedia Appendix 5 provides examples of intervention content to show how findings from the design phases have been incorporated into the platform. This is not an extensive list but rather examples to demonstrate knowledge transmission.

Example 1: Using specific color schemes and animation styles to create vibrant, engaging, and relatable content.

**Figure M1.** Exert from psychoeducational video on helpful and anxious thoughts.


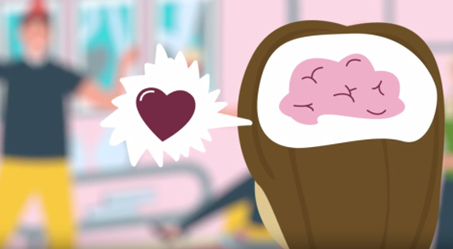


**Figure M2**. Exert of psychoeducational video on the relations between situations, bodily sensations, and actions.


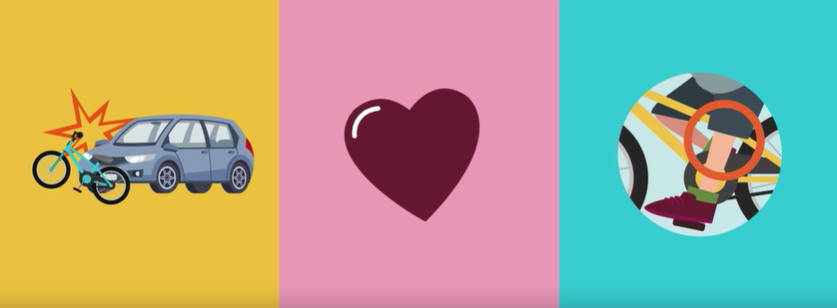


Example 2: Using graphics to illustrate core CBT principles.

**Figure M3.** Illustration of how the brain reacts when meeting a feared stimulus (catastrophizing) leading to avoidance.


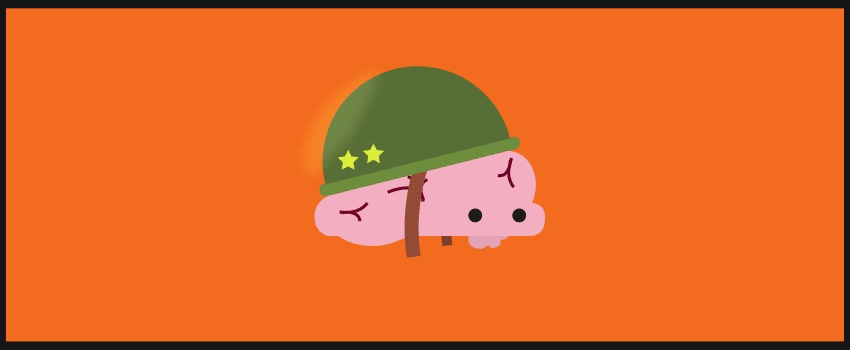


**Figure M4.** Illustration of how thoughts (distorted thinking) alters your perception.


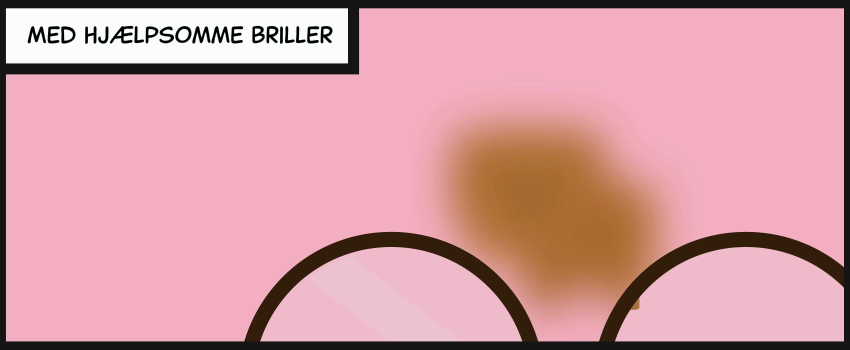

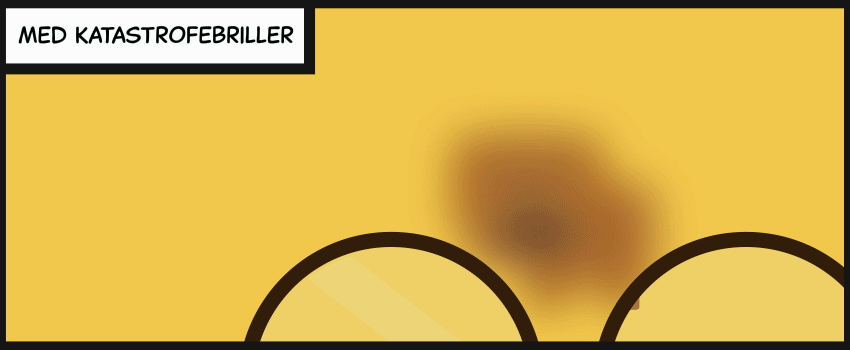


**Figure M5.** Illustration of distorted thinking (reading minds).


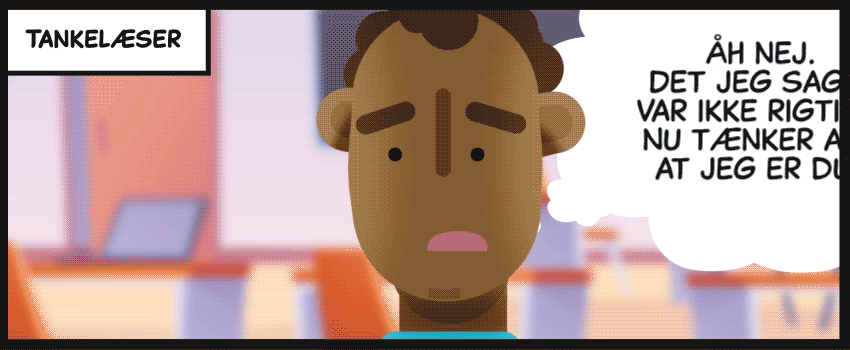


**Figure M6**. Illustration of habituation in exposure.


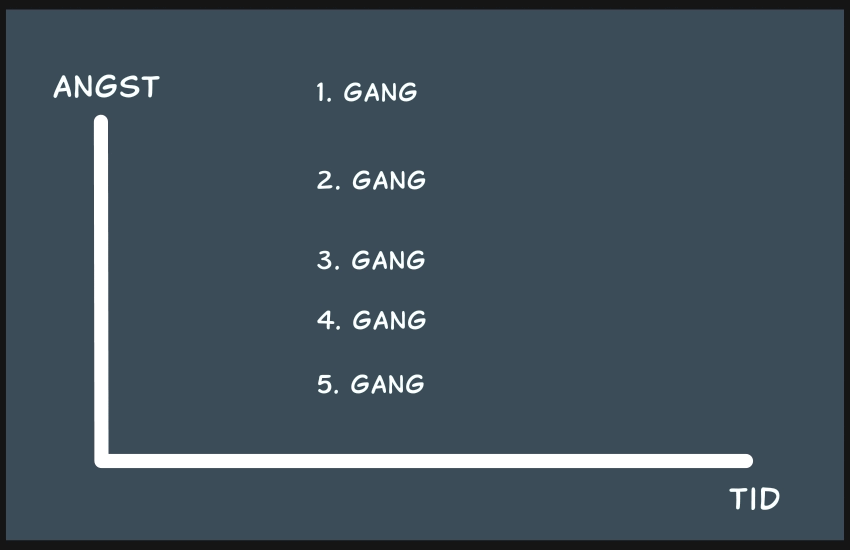


Example 3: Recurring and relatable characters, iGuides, that guide the adolescent through the intervention content using a storytelling approach in a cartoon-like format.

**
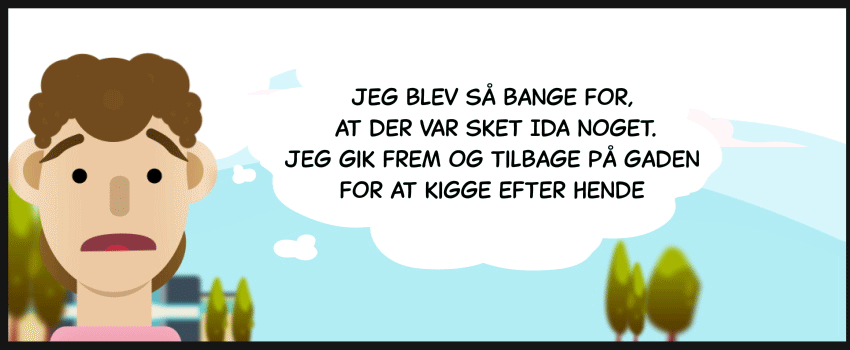

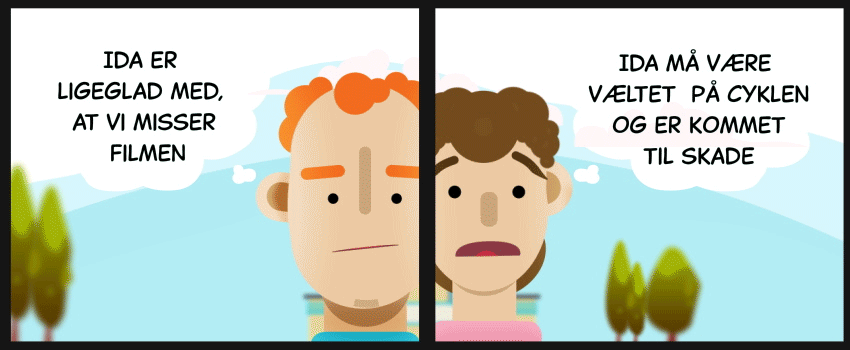
**
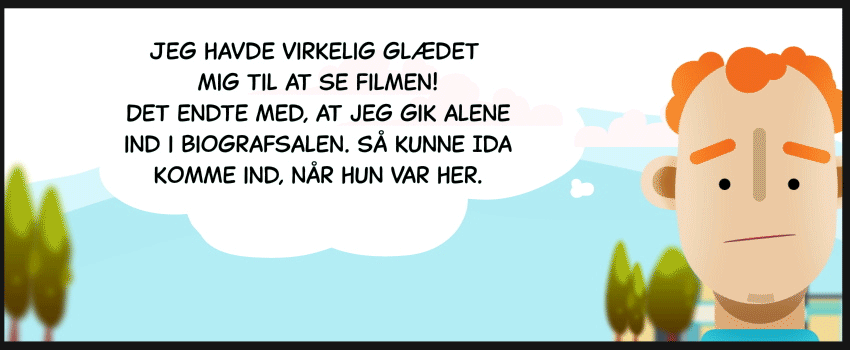
**Figure M7.** Providing relatable examples through a cartoon-like story-telling approach using recurring characters.

**Figure M8.** Using a story-telling approach in a cartoon-like format to explain the principles of exposure, i.e., going to a birthday party on the top floor of a tall buliding when you’re afraid of heights.


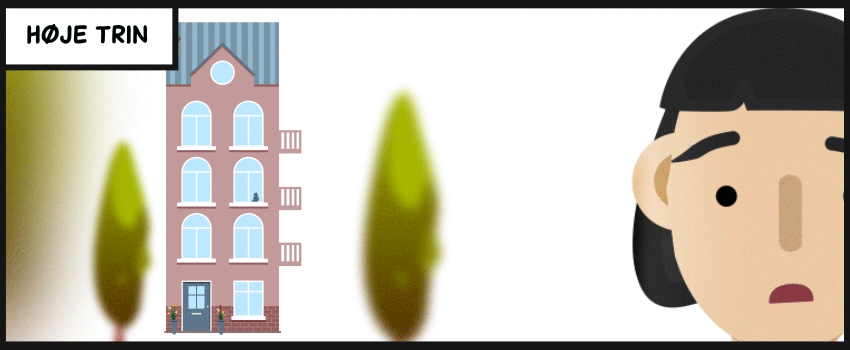

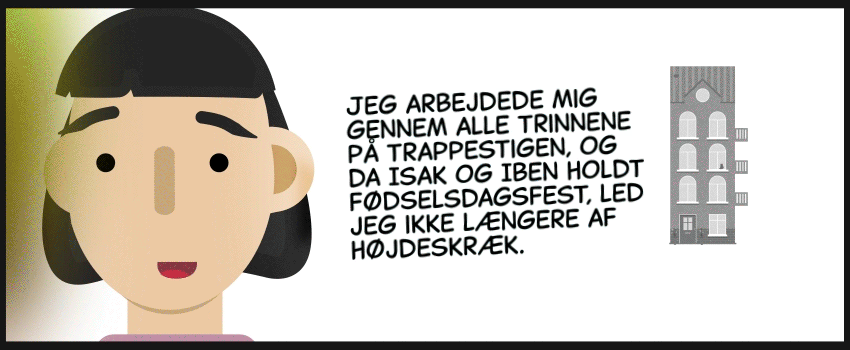

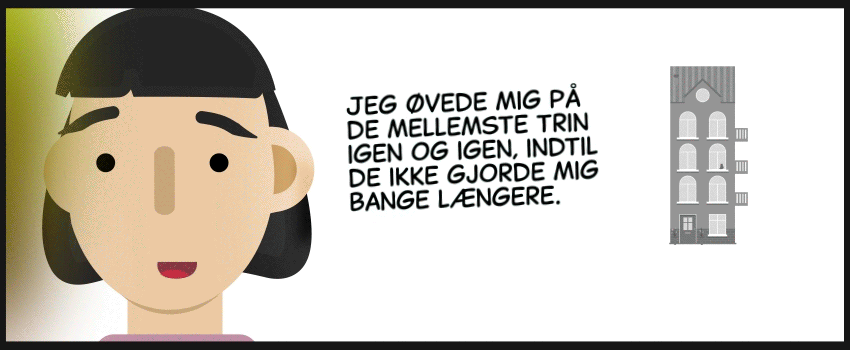


(Continued on next page)


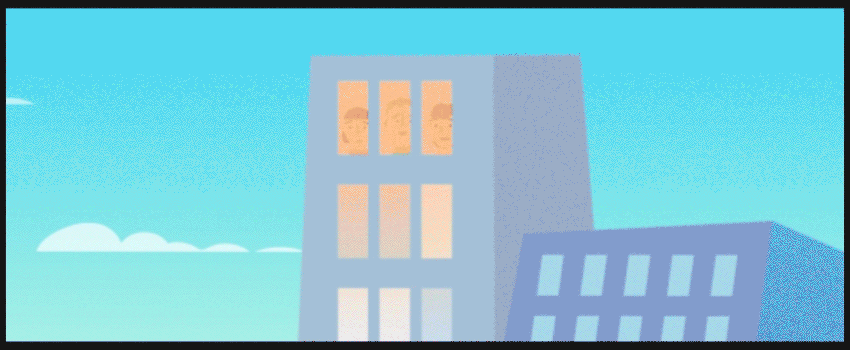


Example 4: Using relatable examples from everyday situations.

**Figure M8.** An example of how anxious (distorted) thoughts may lead you to avoid certain situations, i.e., not raising your hand due to social anxiety.


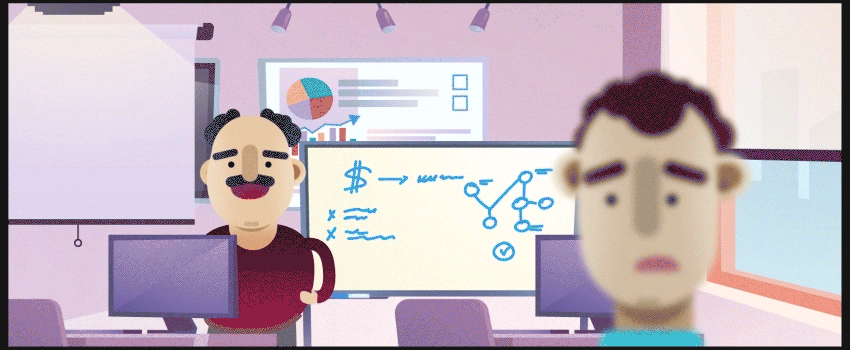

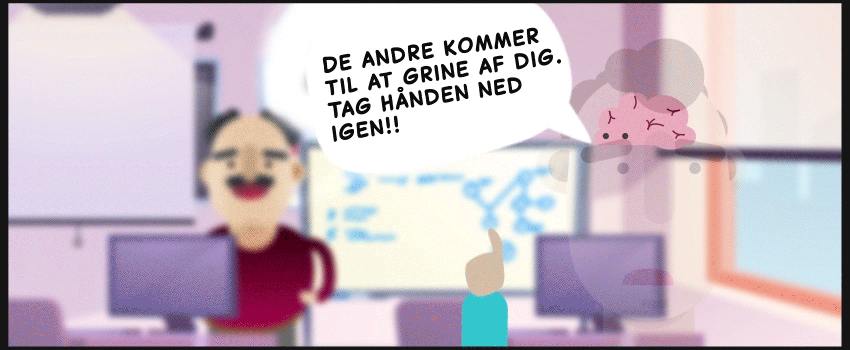

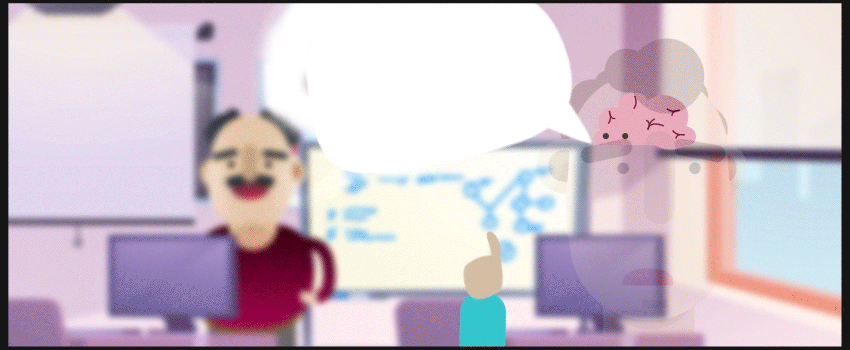


Example 5: Screenshots from the program

**Figure M9.** Screenshots from three different sessions showing the variety of modalities used to disseminate the CBT principles and techniques.


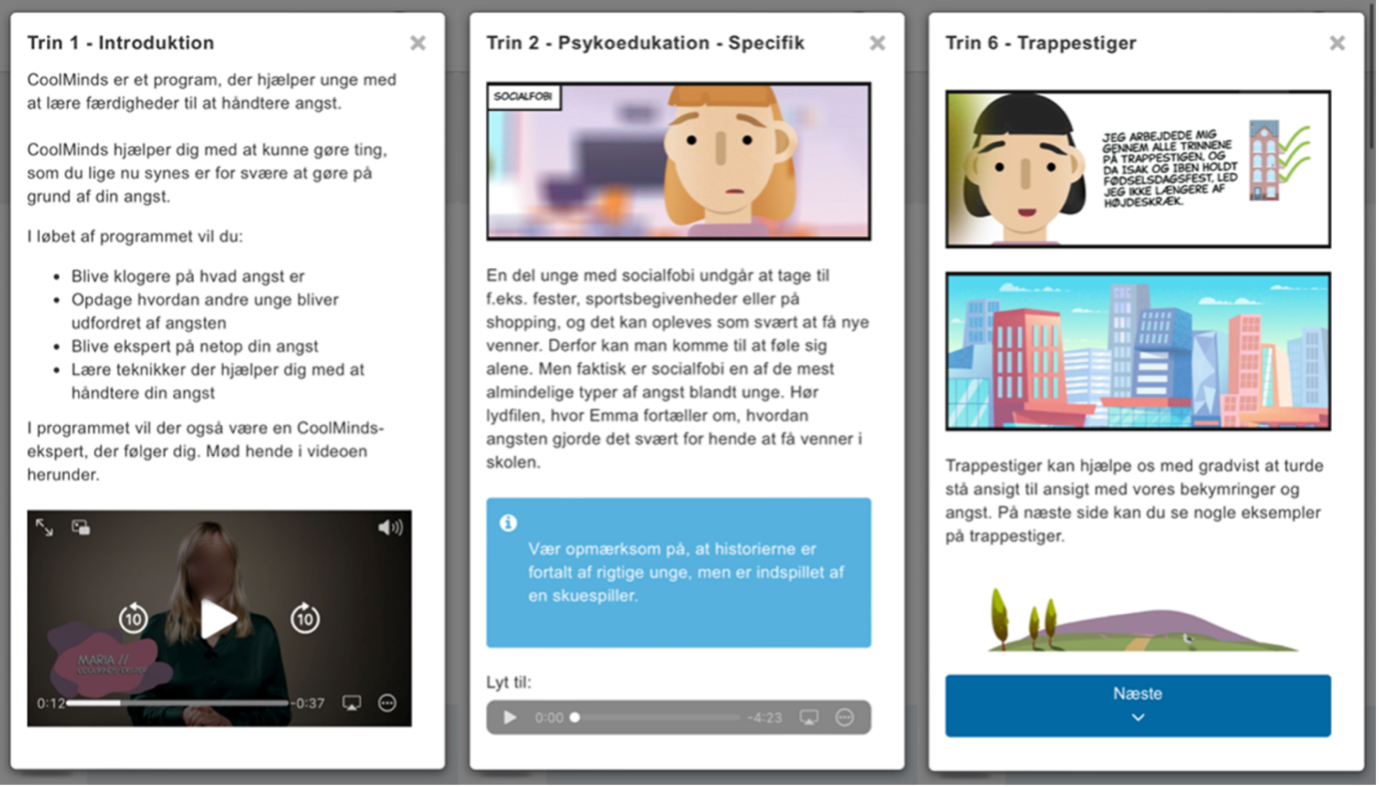

Supplement: Multimedia Appendix 5 [file formative_v9i1e66966_app5.docx]
